# Preliminary Evaluation of the Diagnostic Usefulness of Uroplakin 2 with an Assessment of the Antioxidant Potential of Patients with Bladder Cancer

**DOI:** 10.1155/2018/8693297

**Published:** 2018-11-29

**Authors:** M. Matuszewski, B. Szymańska, A. Długosz, B. Małkiewicz, J. Dembowski, A. Piwowar

**Affiliations:** ^1^Department of Urology and Oncological Urology, Wroclaw Medical University, Wroclaw, Poland; ^2^Department of Toxicology, Wroclaw Medical University, Wroclaw, Poland

## Abstract

**Background:**

Urothelial carcinoma is the most common type of bladder cancer (BC). It makes up more than 90% of all bladder cancers. Uroplakins are tissue-specific, glycoproteins, playing a role in the construction and function of urothelium. The emergence of uroplakins in the urine and/or plasma may be of potential importance in the early detection of BC. In our study, the diagnostic value of plasma and urine uroplakin 2 (UP2) concentration in bladder cancer was investigated, with an assessment of the antioxidant potential of BC patients. The correlation between UP2, total antioxidant capacity (TAC), and concentration of glutathione (GSH) was also examined.

**Materials and Methods:**

This study included 61 BC patients and 33 healthy controls. UP2 concentration was estimated by the immunoenzymatic method (ELISA). TAC and GSH were determined in spectrophotometrically methods.

**Results:**

UP2 concentration in BC patients was significantly higher (p≤0.001) both in plasma and in urine compared to the control groups (C). TAC concentration in urine (p≤0.001) and GSH concentration in plasma (p=0.047) were significantly lower in BC group compared to the C group. The high specificity and sensitivity for UPK2 in plasma (76%, 80%, respectively) and urine (88%, 84%, respectively) were observed. Positive correlations were observed between concentration of UP2 in plasma and TAC concentration in urine and between UP2 concentration in plasma and GSH concentration in the same material.

**Conclusion:**

The study showed the early diagnostic value of urine and plasma UP2 in BC. There was a decrease in UP2 concentration in the urine of patients with the development of BC. The decrease of antioxidant systems (TAC, GSH) indicates their relationship with the BC process. Based on the obtained results, it is justified to continue the study in a larger group of patients with BC.

## 1. Background

Urothelium is a multilayer membrane, which covers renal pelvis, ureters, bladder, and proximal part of urethra and is in direct touch with urine. Urothelium is built with dimensional, asymmetric plaques, whose specific structure is crucial for tightness and elasticity of urothelium. This feature protects bladder walls from rupture by filling bladder with urine. Integral components of these plaques are uroplakins (UP), differential, hexagonal configured, and mutually conjugated proteins. A few isoforms of UP are known in humans: UP1a, UP1b, UP2, UP3a, UP3b, and UP3c. UP can be classified into two main groups: tetraspanins like UP1a and UP1b which have four transmembrane domains and uroplakins with one transmembrane domain: UP2 and isoforms of UP3. Uroplakins form heterodimers in which UP1a is coupled with UP2, and UP1b with UP3 [1–3].

UP2 with a mass of 15kDa is the smallest UP. It is synthetized as a prepro-UP2 with a mass of 19 kDa. In contrast to other UPs, a mature form of UP2 does not consist of any sugar moiety; however its precursor undergoes glycosylation in endoplasmic reticulum (ER). Glycosylation of immature UP2 seems to be crucial in building the dimer with UP1a. UP2 is essential in forming of uroplakins crystals, out of which plaques are built. This status is proofed by the absence of these structures in cells without UP2 coding gene [2, 4].

Disorder of proper expression of UP is connected with pathogenesis of infections, neoplasms of urinary tract, and primary vesicoureteral reflux [1]. Most of bladder cancers (BC) are urothelial neoplasms. In this type of tumors, UP can be released in a greater amount than in regular cases, due to destruction of urothelium. Parallel to tumor growth, physiological urothelial tissue is replaced by cancer cells, with the loss of UP production ability in malignancy process [5, 6].

Development of BC is related to long-term exposition to environmental risk factors, such as smoking, chemical substances in workplace and place of residence (e.g., polycyclic aromatic hydrocarbons: PAHs, aromatic amines, heavy metals, nitrosamines, and pesticides) [7]. Metabolism of these substances is related to an increased production of reactive oxygen species (ROS) and induction of oxidative stress (OS). The role of OS in initiation, promotion, and progression of BC has been described in literature [8]. Elimination of ROS is mainly made by antioxidative systems; therefore its evaluation in BC seems to be essential.

Total antioxidant capacity (TAC) is composed of antioxidants (e.g., ascorbic acid, *α*-tocopherol, *β*-carotene, reduced glutathione, uric acid, and bilirubin) and can be measured in body fluids. TAC level affects intensification and occurrence of OS, responsible for many diseases,* inter alia*, cancers. The content of individual antioxidants in body fluid does not always reflect its real effectiveness. Due to this fact, fast and noninvasive methods of TAC measurement have been elaborated [9–11].

One of the antioxidants, which plays essential role in cell defense from negative effect of oxygen free radicals, is glutathione (GSH). This tripeptide has a capability in H_2_O_2_ and other ROS deactivation and in chelation of heavy metal ions, which participate in ROS forming. GSH conjugates xenobiotics and participates in regeneration of other antioxidants and also ingredients of cell membrane as well as nucleic acids. The role of GSH in apoptosis and cell differentiation is known [12]. GSH occurs in two forms: reduced (GSH) and oxidized (GSSG), but reduced form dominates. It is assumed that reduced level of GSH can be related to development and progression of many cancers due to the higher susceptibility to free radicals [13, 14]. So far GSH was not analyzed in BC patients.

The aim of this study was to evaluate diagnostic value of UP2 in BC, measured in urine and plasma of BC patients, with different tumor stage and grade. This value has not been investigated before. The evaluation of TAC in BC patients urine and measurement of reduced GSH in patients plasma, which was performed to estimate the efficiency of antioxidative processes in patients with ongoing neoplastic process in relationship to changes in UP2 level, were an additional novelty of the study. Moreover, mutual relations between UP2 and other parameters like uroplakin 3a (UP3a), 8-hydroxy-2'deoxyguanosine (8-OHdG), and glutathione S-transferase isozyme-*π* (GST-*π*), which has been previously published, were investigated in the same patients group [15].

## 2. Materials and Methods

### 2.1. Patients

The study group was 61 BC patients of Urology and Oncological Urology Department (Wroclaw Medical University). The group consisted of 51 men (84%) and 10 women (16%), and mean age was 66 years (41-88). All patients were informed about aim of study, participation was voluntary, and all signed written informed consent. The control group included 33 healthy volunteers: 28 men (85%) and 5 women (15%) aged 54-81 years (mean age 65). The characteristics of groups are given in Table 1.

Controls were selected from participants with no history of cancer or other chronic inflammation, which was excluded by clinical examination of the cytology of urine sediment and a urine strip test. The BC patients and subjects from the control group were of similar socioeconomic status. There were no significant differences between these groups. All participants were informed of the aim of the study and gave written consent to participate. The study was approved by Ethics Committee of Wroclaw Medical University (KB-292/2-16).

Based on histopathological examination of tissues (performed in Department of Pathomorphology and Oncological Cytology, Wroclaw Medical University), patients were divided into subgroups, according to tumor stage T (TNM: Tumor Nodules Metastases, 2002r.) and grade (low grade/high grade, WHO/International Society of urological Pathology, ISUP System 2004r.) (Table 1).

### 2.2. Materials

The materials for laboratory tests were human blood and urine. In the BC group, the materials were obtained one day before surgical and any pharmacological treatment. The morning urine samples were collected in polystyrene containers (Aptaca, Italy) and next centrifuged for 10 minutes (1438xg at 4°C), and the obtained supernatant was removed to Eppendorf tubes and stored at -80°C for further investigation. Blood samples were collected into the plastic tubes (BD Vacutainer, with an anticoagulant trisodium citrate buffer, USA). The tubes were centrifuged by MPW-350 laboratory centrifuge (MPW Instruments, Poland) at 1438xg for at least 10 min at 4°C. The supernatant (plasma) was frozen at -80°C until analyzed.

### 2.3. Methods

#### 2.3.1. UP2

UP2 level was measured in urine and plasma by immunoenzymatic (ELISA) Enzyme-Linked Immunosorbent Assay Kit (USCN Life Science Inc., People's Republic of China, by design of Cloud-Clone Corp., USA) according to the manufacturer's instructions in listed test. The microplate has been precoated with an antibody specific to UP2. Standards or samples (100*μ*l) were added to the appropriate microplate wells with a biotin-conjugated antibody specific to UP2. Next, avidin conjugated to Horseradish Peroxidase (HRP) was added to each microplate well and incubated (2h, 37°C). Next TMB (3,3′,5,5′-tetramethylbenzidine) substrate solution was added, which caused that only those wells that contain UP2, biotin-conjugated antibody, and the enzyme-conjugated avidin displayed a change in the color. The enzyme-substrate reaction was terminated by the addition stop solution and the color change was measured spectrophotometrically at a wavelength of 450nm by Synergy HTX Multi-Mode Microplate Reader (BioTek Instruments, Germany). The concentration of UP2 in the samples was then determined by reading the absorption of the samples to the standard curve.

#### 2.3.2. TAC and GSH

TAC and GSH levels were determined spectrophotometrically using the Antioxidant Assay Kit (Cayman Chemical, USA) and Glutathione Assay Kit (Cayman Chemical, USA), respectively, according to the manufacturer's instructions in listed test.

#### 2.3.3. TAC

This assay quantifies the ability of the sample to inhibit an oxidation assay and compares the degree of inhibition by urine to known quantities of Trolox. It relies on the ability of low molecular weight antioxidants in urine to inhibit oxidation of 2, 2′-azino-di-[3-ethylbenzthiasoline sulphonate] (ABTS) to ABTS^+^ by metmyoglobin. Inhibition of absorption at 750 nm is measured by Synergy HTX Multi-Mode Microplate Reader (BioTek Instruments, Germany) and compared to that of the water soluble tocopherol analog, Trolox. The data therefore reflect the net antioxidant capacity of proteins (*e*.*g*., albumin) and small molecules (*e*.*g*., GSH, vitamin E, and vitamin C) normally present in urine [16].

#### 2.3.4. GSH

GSH assay utilizes a carefully optimized enzymatic method, using glutathione reductase, for the quantification of GSH. The sulfhydryl group of GSH reacts with DTNB (5,5′-dithio-bis-2-(nitrobenzoic acid) and produces a yellow colored 5-thio-2-nitrobenzoic acid (TNB). The mixed disulfide, GSTNB (the disulfide product of reaction of GSH with DTNB) that is concomitantly produced, is reduced by glutathione reductase to the GSH and produces more TNB. The rate of TNB production is directly proportional to this reaction which is in turn directly proportional to the concentration of GSH in the sample. Measurement of the absorbance by Synergy HTX Multi-Mode Microplate Reader (BioTek Instruments, Germany) of TNB at 405-414 nm provides an accurate estimation of GSH in the sample [17].

The obtained urinary markers values were calculated in relation to the urine creatinine level previously estimated by Jaffe's routine method [18].

## 3. Statistical Analysis

Statistical analysis was conducted with Statistica PL software (version 13.1). The normality of distribution was checked with the Lilliefors and the Kolmogorov-Smirnov tests. Student's t-test for parametric data and the Mann-Whitney U test for nonparametric data were used. The values of p<0.05 were considered as statistically significant. The associations between continuous variables were analyzed by the Spearman for nonparametric data and Pearson for parametric data. The receiver operating characteristic (ROC) curves were conducted and estimated. The area under the curve (AUC) and the best cut-off point were calculated employing ROC analysis which evaluated the relation between sensitivity and specificity of examined markers.

## 4. Results

The mean plasma concentration of UP2 in BC group was 1.4 higher than in the control group (p≤0.001) (Table 2(a)), whereas the mean urine concentration of UP2 in group of patients with BC was 3.4 higher than in the control group (p≤0.001) (Table 2(b)).

There were no statistically significant differences of UP2 concentration both in plasma or in urine between nonmuscle invasive bladder cancer (NMIBC) and invasive bladder cancer (MIBC) but in both groups UP2 concentration was higher than in the control C (p≤0.001). Similar trend of results was obtained in LG and HG groups (Tables 2(a) and 2(b)). Nonsignificant differences between UP2 concentration in smokers and no-smoking patients were also observed, both in urine and in plasma (Tables 2(a) and 2(b)). Only between BC men and BC women difference was significant in plasma UP2 concentration (p≤0.001) (Table 2(a)), while any significant difference between the concentrations of UP2 in the plasma of women and men in the control group was found (p>0.05).

A low positive correlation between the UP2 excreted into urine and age and higher correlation between UP2 in plasma and sex were noted only in patients group (Table 6).

**Figure 1:**
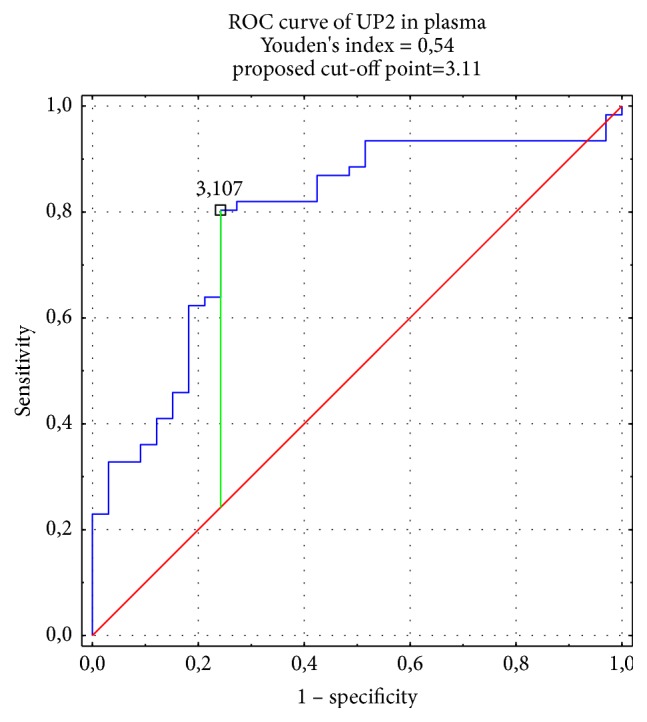
ROC curve of UP2 in plasma.

**Figure 2:**
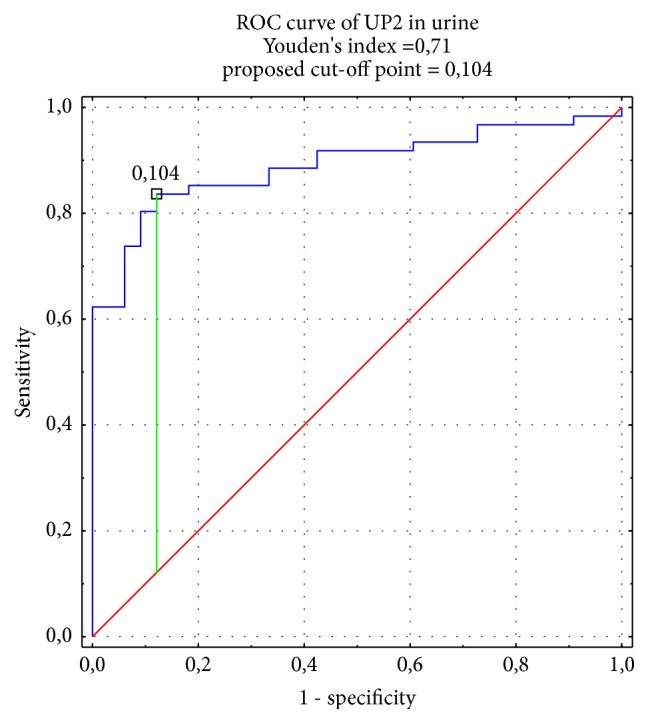
ROC curve of UP2 in urine.

The diagnostic value of the examined tested UP2 in both biological fluids was evaluated. The high specificity and sensitivity for UP2 in plasma (76%, 80%, respectively) and urine (88%, 84%, respectively) were observed. The results show that the concentration of UP2 measured by immunoenzymatic methods has a good diagnostic value in BC group. The AUC was calculated as 0.79 in plasma (Figure 1) and 0.89 in urine (Figure 2). It points a good diagnostic value of UP2 in plasma or in urine (over 0.8). The designated cut-off points were for UP2 in plasma 3.107 ng/mL and for UP2 in urine 0.104 ng/mg cr., respectively.

The mean TAC concentration in urine in BC group was 1.6 lower than in control group C (p≤0.001) (Table 3). TAC values were also higher for MIBC compared to NMIBC, and in the LH than HG groups, but not statistical significantly. In both groups (MIBC and NMIBC) TAC was significantly lower than in the C group (p≤0.001). A similar trend was obtained in LG and HG groups than in the C group (p≤0.001). There were no significant differences in examined TAC concentrations between BC men and women or between BC smokers and nonsmokers (Table 3).

The concentration of GSH in plasma was significantly lower (about 1.2) in BC patients than in controls (p=0.047) (Table 4). No significant differences in GSH concentration in urine were observed in comparison of NMIBC and MIBC, either between LG and HG groups. The GSH concentration in HG group was significantly lower than in the C group (p=0.025). The differences between smoking or nonsmoking BC, women or men, were also not statistically significant (Table 4).

Connection between the determined parameters was presented in Table 5. Positive, but not strong correlations were observed between UP2 (plasma) and TAC (urine) and between UP2 (plasma) and GSH (plasma). No significant correlation was noted between UP2 (plasma) and UP2 (urine) and between TAC (urine) and GSH (plasma).

Additionally, correlations between UPK2, TAC, GSH, and other parameters examined earlier were investigated. These parameters included UP3a, also 8-OHdG, specific for oxidative DNA damage marker, and GST*π*, detoxification enzyme located in the urothelium. The high correlation between UP2 (urine) with UP3 (urine), lower with 8-OHdG (urine), and isoenzyme GST*π* (urine), were observed (Table 6). The correlations observed in urine were not reflected in the resultants obtained patients in plasma.

## 5. Discussion

UP3 was the first uroplakin which was evaluated for usefulness in BC diagnostics. The antibody used in this study was characterized with high specificity, but its sensitivity (10-60%) was not sufficient. In 2014 Smith et al. [19] have published the study with the comparison of monoclonal antibody used for UP3 detection with the new (at that time) UP2 binding antibody. To perform this study, they have detected both uroplakins (UP2 and UP3) in tissue samples of BC. Patients with different neoplasm of urinary tract have participated in this study. In patients with bladder neck tumor, UP2 antibody has shown higher sensitivity (63% UP2; 19% UP3) with a slightly lower specificity (95% UP2; 100% UP3). Similar results have been obtained for upper urinary tract urothelial carcinoma (UUTUC), 68% of sensitivity for UP2 and 23% for UP3 and 100% specificity for both uroplakins. In metastases of urothelial carcinoma also higher sensitivity of UP2 (73%) than UP3 (37%) was noted. In histopathological samples of nonurothelial cancers uroplakins have not been detected. Moreover, it has been noted that UP3 was present only in cell cytoplasmic membrane, and UP2 was detected in cell membrane as well as in cytoplasm [19].

Occurrence frequency of UP2 and UP3 in several urothelial cancers has been also investigated by Li et al. [20]. In this study, histological tumor samples of patients with various urothelial cancers (UCs) variants, including 105 conventional bladder Ucs (BUCs), 90 UUTUCs, 47 micropapillary, 16 plasmacytoid, 22 small cell carcinoma, and 41 sarcomatoid UC variants, were collected. Obtained results have been similar to those presented by S.C. Smith [19] and showed that in most of UC, UP2 was more often present in tumor cells than UP3.

Our study showed that measurement of UP2 in urine as well as in plasma can be used in BC diagnostics, because plasma and urine level of this protein were statistically significantly higher in BC patients (p≤0.001) than in the control group. So far similar studies have not been published, and UP2 expression was investigated only in histopathological BC samples with immunohistochemical methods. A low UP2 concentration (especially in urine) was the possible cause of this condition. However, the use of sensitive test (detection in ng/ml) has allowed us to measure UP2 concentration in body fluids (urine, plasma).

In the performed study, we observed that, together with higher tumour stage and grade (MIBC, HG), urine concentration of UP2 decreases. However, this UP2 fall was not observed in plasma and average UP2 concentration in MIBC and NMIBC as well as in LG and HG groups was similar. High, statistically significant differences between mean UP2 concentrations in LG tumors and NMIBC, both in plasma and in urine, in comparison to the control group (p≤0.001) suggest that UP2 can be a potential marker in the early diagnostics of BC. Early detection of BC increases the possibility of proper treatment and improves patients' prognosis.

In literature, similar type of relationship was described in reference to UP2 expression in BC tissue. Histopathological samples obtained from patients with tumor of higher malignancy level have lower membrane expression of UP2 [21]. In the study of Vargi et al. [22] on UP1b, it has been demonstrated that hypermethylation of CPG sites presented close to promoter of this protein coding gene results in silencing of this gene. There exists a correlation between the expression of DAPK1 protein (death-associated protein kinase 1) promoter, which often undergoes hypermethylation, and expression of UP2 coding gene [23]. It suggests that similar mechanism may occur in UP2 as in UP1b.

In MIBC and NMIBC as well as in LG and HG tumors, any statistically significant differences were observed. The lack in significance could be caused by a small group of patients, especially with MIBC (n=15). The study on larger patients' group could be evaluated if noted a downward trend of UP2 level in step when disease progression is proper. If this correlation is factual, UP2 in urine could be used not only as diagnostic marker of BC, but also as a prognostic parameter in disease monitoring. The study has not shown the correlation between UP2 in plasma and in urine. Statistically higher concentration of UP2 (p≤0.001) in men plasma than women with BC, and the lack of such a correlation between healthy men and women, could suggest the involvement of other mechanisms, which regulate the increase of UP2 expression in urothelium or more intensive process of its release to blood. However these data need to be evaluated and analyzed in detail.

High specificity and sensitivity of UP2, shown in our study (76% and 80% in plasma and 88% and 84% in urine), point on its high value in BC diagnostics.

Disorders in oxidative and antioxidative balance occur in neoplastic processes [8, 24–26]. TAC has been evaluated in many diseases, also in BC, but only in patients plasma [27]. In available literature, there are not any studies on TAC in urine of BC patients. Our study has shown relevant lower TAC activity in BC patients urine, almost 40% lower than in control group. An important decrease of TAC activity has been observed in all BC stages (LG, HG, MIBC, and NMIBC) in comparison to control (p≤0.001). However, any relevant differences between lower and higher BC stages were noted. We observed the correlation between UP2 both in urine and in plasma and level of TAC activity in urine.

A decrease of antioxidative systems activity in BC shows on its depletion and suggests that supplementation with egzogenic antioxidants, such as vitamins, can be therapy complement. Such conclusion has been presented by the cohort study of Nechuta et al. [28]. In the group of oncological patients treated with vitamin supplements, authors have observed 18% lower risk of death in comparison to patients without supplementation. Also chance of disease recurrence was lower of almost 22%. It has been established that antioxidants can inhibit growth of existing neoplasm, among others by activation of macrophages and lymphocytes, which have cytotoxic effect on tumor cells, regulate p53 gene expression, and restrain angiogenesis especially by vitamin E, carotene, and glutathione [12].

Disorders in glutathione homeostasis are considered as one of risk factors of many diseases, also cancers. It is supposed that lower GSH level may be related to cancer development and progression due to an increased cell sensitivity on free radicals [14]. Moreover, it has been demonstrated that GSH prevents activation of NF-*κ*B (*nuclear factor kappa-light-chain-enhancer of activated B cells*) responsible for i.a. apoptosis inhibition [29, 30]. This thesis has not been confirmed in other studies, which shows elevated GSH level and enzymes involved in GSH synthesis in neoplastic cells [31]. Our study shows significantly lower GSH level in BC patients (p=0.047) in comparison to the control group. GSH concentration in group of patients with HG tumor was also significantly lower (p=0.025) with reference to the control. It points out a decrease of GSH concentration in BC patients due to ongoing neoplastic process. So far there has been the lack of data for this parameter measurement in BC. Any significant differences between GSH level according to tumor grade and stage (p>0.05) were observed.

Results obtained by other researchers show significant role of GSH in neoplastic growth [12, 32]. The study of Lash et al. [33], made on cell culture of prostate cancer, shows that more malignant cells have 4.2 higher GSH level, and they were more resistant to chemotherapy. Research made by Byun et al. [34] on cell culture T24 points on relation between cancer cell GSH level and cisplatin-resistant. It confirms that GSH could be a useful indicator of ongoing neoplastic process, especially during chemotherapy efficiency monitoring. Elevated GSH level in cancer cell is induced by defence mechanisms: protection against immunological response, cytostatic resistance, and radiotherapy [35, 36]. The study of Safarinejad et al. [37] on role of glutathione transferase (GST) polymorphism genes in urothelial cancer development shows that some of gene variants coding glutathione S-transferase isozyme-*π*-1 (GSTP1) were related to higher risk of getting BC. Moreover, BC patients with this genotype were more susceptible to progression of more invasive and more malignant form of BC.

BC is classified as environmentally related. Higher risk of BC development is observed in tobacco smokers and in people exposed to chemical carcinogens (e.g., aromatic amines, PAHs, heavy metals, and nitrosamines). These factors are related to the production of free radicals and oxidative stress, which is one of BC carcinogenesis factors [7]. Search on noninvasive diagnostic markers and role of oxidative stress in BC development are the aim of our researches for few years [38, 39]. Based on our former study, with the same BC patients group, we have evaluated the relation between plasma and urine UP2 concentration and a marker of oxidative DNA damage, 8-OHdG. In available literature, other authors have described influence of various xenobiotics on increasing 8-OHdG level in monitoring exposition on genotoxic substances. Our study shows the correlation between UP2 and 8-OHdG level in urine, which points to UP2 sensibility on genotoxic and oxidative influence of xenobiotics presented in environment.

Glutathione transferase is an important detoxification enzyme and plays the main role in xenobiotics transformation, especially PAHs. Isozyme GST*π* is particularly involved in carcinogenesis process [40–43]. Our studies showed a significantly higher GST*π* urine concentration in BC patients than in the healthy group. An increased urine concentration of UP2 could be related to an extensive destruction of urothelium which leads to UP2 releasing from ruined urothelial cells and also by UP2 production by neoplastic cells. The simultaneous growth of UP2 and GST*π* concentrations in BC is probably related to an increasing amount of tumor cells, able to produce synthesis of this protein [44].

The correlation between former evaluated UP3 and UP2 urine concentration in the study group (R=0.491; p≤0.001) shows relationship between these two uroplakins, which are released to urine due to urothelium damage. We observed (in our studies) a higher plasma concentration of UP2 in relation to urine concentration, but in UP3 this trend was reverse. The possible reason of this situation could be the fact that UP2 is sugar free low molecular weight protein (15kDa) and it penetrates more easily to blood than UP3 [2, 4]. Li et al. [20] point to UP2 as a better expectant marker of BC, because it is often present on tumor cells than UP3. Our preliminary study shows UP2 usefulness in early BC diagnostic and its prognostic value, related to decreasing urine UP2 level in step with cancer growth.

## 6. Conclusion

Our study showed usability of UP2 evaluated in urine and plasma as a potential marker in early BC diagnostics. UP2 prognostic value is evidenced by the decline of its concentration along with BC development, reported in measurement of UP2 in urine. Due to this fact, further researches on UP2 application as a BC marker in larger patients group are indicated. Efficiency decrease of antioxidative systems (TAC, GSH) points to its association with neoplasm and suggests usefulness of vitamin supplementation and/or introduction of diet rich in antioxidants (vegetables, fruits) as a complementation of BC therapy.

## Figures and Tables

**Table 1:** Population characteristic.

Population characteristic	N (%)
PATIENTS	61

Age, y, range (median)	66 (41-88)

Male	51 (84)

Female	10 (16)

Smokers	47 (77)

Non-smoking	14 (23)

*Clinical staging*	

Ta	28 (46)

T1	18 (30)

T2	4 (6)

T3	5 (8)

TIS	6 (10)

*Clinical grading*	

LG	29 (53)

HG	32 (47)

*Clinical subgroups*	

NMIBC	46 (75)

MIBC	15 (25)

CONTROLS	33

Age, y, range (median)	65 (54-81)

Male	28 (85)

Female	5 (15)

Smokers	24 (73)

Nonsmokers	9 (27)

Ta, T1, T2, TIS: subgroups, according to tumor stage T (TNM: Tumor Nodules Metastases, 2002r.); NMIBC: non-muscle invasive BC; MIBC: muscle invasive BC; LG: low grade; HG: high grade; n: number of cases; y: years.

**Table:** (a) UP2 level in the plasma in BC patients and control group

Groups	UP2			p-value
	in plasma [ng/mL]			
	Me	Mean	SD	in plasma
BC	3.65	3.71	1.29	p≤0.001 BC vs C
C	2.63	2.72	0.68	

NMIBC	3.55	3.73	1.40	p=0.650 NMIBC vsMIBC
				p≤0.001 NMIBC vs C
MIBC	3.45	3.64	0.90	
				p=0.003 MIBC vs C

LG	3.43	3.60	1.20	p=0.789 LG vs HG
				p≤0.001 LG vs C
HG	3.47	3.83	1.39	
				p≤0.001 HG vs C

M	3.65	3.87	1.34	p≤0.001 M vs W
W	2.97	2.89	0.48	

Non-smoking BC	3.46	3.93	1.98	p=0.659 non-smoking BC vs smoking BC
Smoking BC	3.25	3.66	1.11	

UP2: uroplakin 2; BC: patients group; C: control group; NMIBC: non-muscle invasive BC; MIBC: muscle invasive BC; LG: low grade; HG: high grade; M: men; W: women; SD: standard deviation; p: statistically significant difference; Me: median.

**Table:** (b) UP2 level in the urine in BC patients and control group

Groups	UP2			p-value
	in urine [ng/mg cr.]			
	Me	Mean	SD	in urine
BC	0.24	0.50	0.58	p≤0.001 BC vs C
C	0.07	0.08	0.04	

NMIBC	0.26	0.54	0.64	p=0.939 NMIBC vs MIBC
				p≤0.001 NMIBC vs C
MIBC	0.24	0.38	0.32	
				MIBC vs C p≤0.001

LG	0.25	0.55	0.66	p=0.846 LG vs HG
				p≤0.001 LG vs C
HG	0.26	0.45	0.47	
				p≤0.001 HG vs C

M	0.24	0.50	0.62	p=0.386 M vs W
W	0.56	0.49	0.31	

Non-smoking BC	0.64	0.85	0.90	p=0.640 non-smoking BC vs smoking BC
Smoking BC	0,24	0.43	0.46	

UP2: uroplakin 2; BC: patients group; C: control group; NMIBC: non-muscle invasive BC; MIBC: muscle invasive BC; LG: low grade; HG: high grade; M: men; W: women; SD: standard deviation; p: statistically significant difference; Me: median.

**Table 3:** TAC level in the urine in BC patients and control group.

Groups	TAC in urine [mM/mM cr.]		
	Mean	SD	p-value
BC	1.26	0.63	p≤0.001 BC vs C
C	2.05	0.46	

NMIBC	1.26	1.23	p=0.484 NMIBC vs MIBC
MIBC	1.36	0.60	

LG	1.22	0.54	p=0.621 LG vs HG
HG	1.33	0.73	

M	1.28	0.65	p=0.733 M vs W
W	1.20	0.57	

Non-smoking patients	1.36	0.56	p=0.593 non-smoking vs smoking
Smoking patients	1.24	0.65	

BC: patients group; C: control group; NMIBC: non-muscle invasive BC; MIBC: muscle invasive BC; LG: low grade; HG: high grade; M: men; W: women; SD: standard deviation; p: statistically significant difference.

**Table 4:** GSH level in the plasma in patients BC and control group.

Groups	GSH in plasma [*μ*M]		
	Mean	SD	p-value
BC	5.14	2.02	p=0.047 BC vs C
C	6.04	2.05	

NMIBC	5.23	2.08	p=0.451 NMIBC vs MIBC
MIBC	4.85	2.08	

LG	5.31	2.42	p=0.507 LG vs HG
HG	4.95	1.62	

M	5.32	2.05	p=0.129 M vs W
W	4.23	2.06	

Non-smoking patients	4.66	2.45	p=0.403 non-smoking vs smoking
Smoking patients	5.25	1.99	

BC: patients group; C: control group; NMIBC: non-muscle invasive BC; MIBC: muscle invasive BC; LG: low grade; HG: high grade; M: men; W: women; SD: standard deviation; p: statistically significant difference.

**Table 5:** Correlations between UP2 and parameters of antioxidative status in BC group.

**R**	UP2 (urine)	UP2 (plasma)	GSH (plasma)	TAC (urine)
UP2 (urine)	-	0.206	-0.081	-0.105

UP2 (plasma)	0.206	-	0.278*∗*	0.293*∗*

GSH (plasma)	-0.081	0.278*∗*	-	0.221

TAC (urine)	-0.105	0.293*∗*	0.221	-

*∗*: positive significant correlations between markers.

**Table 6:** Correlations between UP2 and age, sex, and other markers in BC group.

Correlations UP2 in group of BC	R	p
UP2 (urine) vs age	0.296	0.021
UP2 (plasma) vs sex	0.420	≤0.001
UP2 (urine) vs UP3a (urine)	0.491	≤0.001
UP2 (urine) vs 8-OHdG (urine)	0.331	0.009
UP2 (urine) vs GST*π* (urine)	0.364	0.019
UP3a (plasma) vs TAC (urine)	-0.476	≤0.001

R: Spearman correlation coefficient; p: level of significance.

## Data Availability

The data used to support the findings of this study are available from the corresponding author upon request.

## References

[1] Lis J., Kątnik-Prastowska I., Tupikowski K., Matejuk A. (2015). Uroplakins as markers of diseases of the urinary system. *Postępy Higieny i Medycyny Doświadczalnej*.

[2] Desalle R., Chicote J. U., Sun T.-T., Garcia-España A. (2014). Generation of divergent uroplakin tetraspanins and their partners during vertebrate evolution: Identification of novel uroplakins. *BMC Evolutionary Biology*.

[3] Matuszewski M. A., Tupikowski K., Dołowy Ł., Szymańska B., Dembowski J., Zdrojowy R. (2016). Uroplakins and their potential applications in urology. *Central European Journal of Urology*.

[4] Kątnik-Prastowska I., Lis J., Matejuk A. (2014). Glycosylation of uroplakins. Implications for bladder physiopathology. *Glycoconjugate Journal*.

[5] Yuasa T., Yoshiki T., Isono T., Tanaka T., Hayashida H., Okada Y. (1999). Expression of transitional cell‐specific genes, uroplakin Ia and II, in bladder cancer: Detection of circulating cancer cells in the peripheral blood of metastatic patients. *International Journal of Urology*.

[6] Lu J. J., Kakechi Y., Takahashi T., Wu X. X., Yuasa T., Yoshiki T. (2000). Detection of circulating cancer cells by reverse transcription-polymerase chain reaction for uroplakin II in peripheral blood of patients with urothelial cancer. *Clinical Cancer Research*.

[7] Długosz A., Gąsior J., Guzik A. (2015). The influence of environmental risk factors on the development of bladder cancer. *Oncology*.

[8] Sawicka E., Lisowska A., Kowal P., Długosz A. (2015). The role of oxidative stress in bladder cancer. *Postepy Higieny i Medycyny Doswiadczalnej*.

[9] Augustyniak A., Skrzydlewska E. (2004). Antioxidative abilities during aging. *Postępy Higieny i Medycyny Doświadczalnej*.

[10] Grajek W. W. (2004). Role of Antioxidants in Reducing the Occurrence Risk of Cancer and Cardiac Vascular Diseases. *Food: science - technology - quality*.

[11] Gryszczyńska B., Iskra M. (2008). Interaction between exogenous and endogenous antioxidants in the human body. *Nowiny Lekarskie*.

[12] Bilska A., Kryczyk A., Włodek L. (2007). The different aspects of the biological role of glutathione. *Postepy higieny i medycyny doświadczalnej (Online)*.

[13] Pastore A., Federici G., Bertini E., Piemonte F. (2003). Analysis of glutathione: implication in redox and detoxification. *Clinica Chimica Acta*.

[14] Traverso N., Ricciarelli R., Nitti M. (2013). Role of Glutathione in Cancer Progression and Chemoresistance. *Oxidative Medicine and Cellular Longevity*.

[15] Szymańska B., Matuszewski M., Dembowski J., Zdrojowy R., Długosz A. (2018). Uroplakin IIIa Is a Marker in Bladder Cancer but Seems Not to Reflect Chemical Carcinogenesis. *BioMed Research International*.

[16] Jackson R., Ramos C., Gupta C., Gomez-Marin O. (2010). Exercise decreases plasma antioxidant capacity and increases urinary isoprostanes of IPF patients. *Respiratory Medicine*.

[17] Koracevic D., Koracevic G., Djordjevic V., Andrejevic S., Cosic V. (2001). Method for the measurement of antioxidant activity in human fluids. *Journal of Clinical Pathology*.

[18] Mazzachi B. C., Peake M. J., Ehrhardt V. (2000). Reference range and method comparison studies for enzymatic and Jaffe creatinine assays in plasma and serum and early morning urine. *Clinical Laboratory*.

[19] Smith S. C., Mohanty S. K., Kunju L. P. (2014). Uroplakin II outperforms uroplakin III in diagnostically challenging settings. *Histopathology*.

[20] Li W., Liang Y., Deavers M. T. (2014). Uroplakin II is a more sensitive immunohistochemical marker than uroplakin III in urothelial carcinoma and its variants. *American Journal of Clinical Pathology*.

[21] Hoang L. L., Tacha D., Bremer R. E., Haas T. S., Cheng L. (2015). Uroplakin II (UPII), GATA3, and p40 are highly sensitive markers for the differential diagnosis of invasive urothelial carcinoma. *Applied Immunohistochemistry & Molecular Morphology *.

[22] Varga A. E., Leonardos L., Jackson P., Marreiros A., Cowled P. A. (2004). Methylation of a CpG Island within the Uroplakin Ib Promoter: A Possible Mechanism for Loss of Uroplakin Ib Expression in Bladder Carcinoma. *Neoplasia*.

[23] Xie J., Chen P., Zhang J. (2017). The prognostic significance of DAPK1 in bladder cancer. *PLoS ONE*.

[24] Kumar B., Koul S., Khandrika L., Meacham R. B., Koul H. K. (2008). Oxidative stress is inherent in prostate cancer cells and is required for aggressive phenotype. *Cancer Research*.

[25] Saed G. M., Diamond M. P., Fletcher N. M. (2017). Updates of the role of oxidative stress in the pathogenesis of ovarian cancer. *Gynecologic Oncology*.

[26] Reuter S., Gupta S. C., Chaturvedi M. M., Aggarwal B. B. (2010). Oxidative stress, inflammation, and cancer: how are they linked?. *Free Radical Biology & Medicine*.

[27] Mirkheshti M. N., Moval A., Shafian M. (2009). Manganese, chromium and the oxidation status in bladder cancer. *Trace elements and Electrolytes*.

[28] Nechuta S., Lu W., Chen Z. (2011). Vitamin supplement use during breast cancer treatment and survival: a prospective cohort study. *Cancer Epidemiology Biomarkers & Prevention*.

[29] Jones J. T., Qian X., van der Velden J. L. (2016). Glutathione S-transferase pi modulates NF-kB activation and pro-inflammatory responses in lung epithelial cells. *Redox Biology*.

[30] Pordanjani S. M., Hosseinimehr S. J. (2016). The role of NF-*κ*B inhibitors in cell response to radiation. *Current Medicinal Chemistry*.

[31] Estrela J. M., Ortega A., Obrador E. (2006). Glutathione in cancer biology and therapy. *Critical Reviews in Clinical Laboratory Sciences*.

[32] Budzik M. P., Badowska-Kozakiewicz A. M. (2013). Multidrug resistance associated with glutathione. *Menopause Review*.

[33] Lash L. H., Putt D. A., Jankovich A. D. (2015). Glutathione levels and susceptibility to chemically induced injury in two human prostate cancer cell lines. *Molecules*.

[34] Byun S.-S., Kim S. W., Choi H., Lee C., Lee E. (2005). Augmentation of cisplatin sensitivity in cisplatin-resistant human bladder cancer cells by modulating glutathione concentrations and glutathione-related enzyme activities. *BJU International*.

[35] Hayes J. D., Flanagan J. U., Jowsey I. R. (2005). Glutathione transferases. *Annual Review of Pharmacology and Toxicology*.

[36] Coles B. F., Kadlubar F. F. (2003). Detoxification of electrophilic compounds by glutathione S-transferase catalysis: determinants of individual response to chemical carcinogens and chemotherapeutic drugs?. *BioFactors*.

[37] Safarinejad M. R., Safarinejad S., Shafiei N., Safarinejad S. (2013). Association of genetic polymorphism of glutathione S-transferase (GSTM1, GSTT1, GSTP1) with bladder cancer susceptibility. *Urologic Oncology: Seminars and Original Investigations*.

[38] Szymańska Beata, Sawicka Ewa, Guzik Anna, Zdrojowy Romuald, Długosz Anna (2017). The Diagnostic Value of Nuclear Matrix Proteins in Bladder Cancer in the Aspect of Environmental Risk from Carcinogens. *BioMed Research International*.

[39] Szymańska B., Pawlik K. J., Sawicka E. (2017). Evaluation of NMP22 in bladder cancer patients sensitive to environmental toxins. *Advances in Clinical and Experimental Medicine*.

[40] Oğuztüzün S., Sezgin Y., Yazci S., Firat P., Özhavzali M., Özen H. (2011). Expression of glutathione-S-transferases isoenzymes and p53 in exfoliated human bladder cancer cells. *Urologic Oncology: Seminars and Original Investigations*.

[41] Marchewka Z., Piwowar A., Ruzik S., Długosz A. (2017). Glutathione S - transferases class Pi and Mi and their significance in oncology. *Postepy higieny i medycyny doswiadczalnej (Online)*.

[42] Hassan A. A. M., Tagliabue G., Codegoni A. M., D'Incalci M., El-Sewedy S. M., Airoldi L. (1997). Glutathione S-transferase activity and glutathione content in human bladder carcinoma associated with schistosomiasis: Comparison with uninvolved surrounding tissues. *Cancer Letters*.

[43] Savic-Radojevic A., Mimic-Oka J., Pljesa-Ercegovac M. (2007). Glutathione S-transferase-P1 expression correlates with increased antioxidant capacity in transitional cell carcinoma of the urinary bladder. *European Urology*.

[44] Lafuente A., Rodriguez A., Gibanel R. (1997). Limitations in the use of glutathione S-transferase p1 in urine as a marker for bladder cancer. *Anticancer Reseach*.

